# Supplementary figures and images for: Comparison of dose-adjusted EPOCH-R and R-CHOP in diffuse large B-cell lymphoma with high Ki67 expression: Results from a prospective observational study
Source: PLoS One. 2026 May 22;21(5):e0350024. doi: 10.1371/journal.pone.0350024 (PMC13196921; doi:10.1371/journal.pone.0350024)

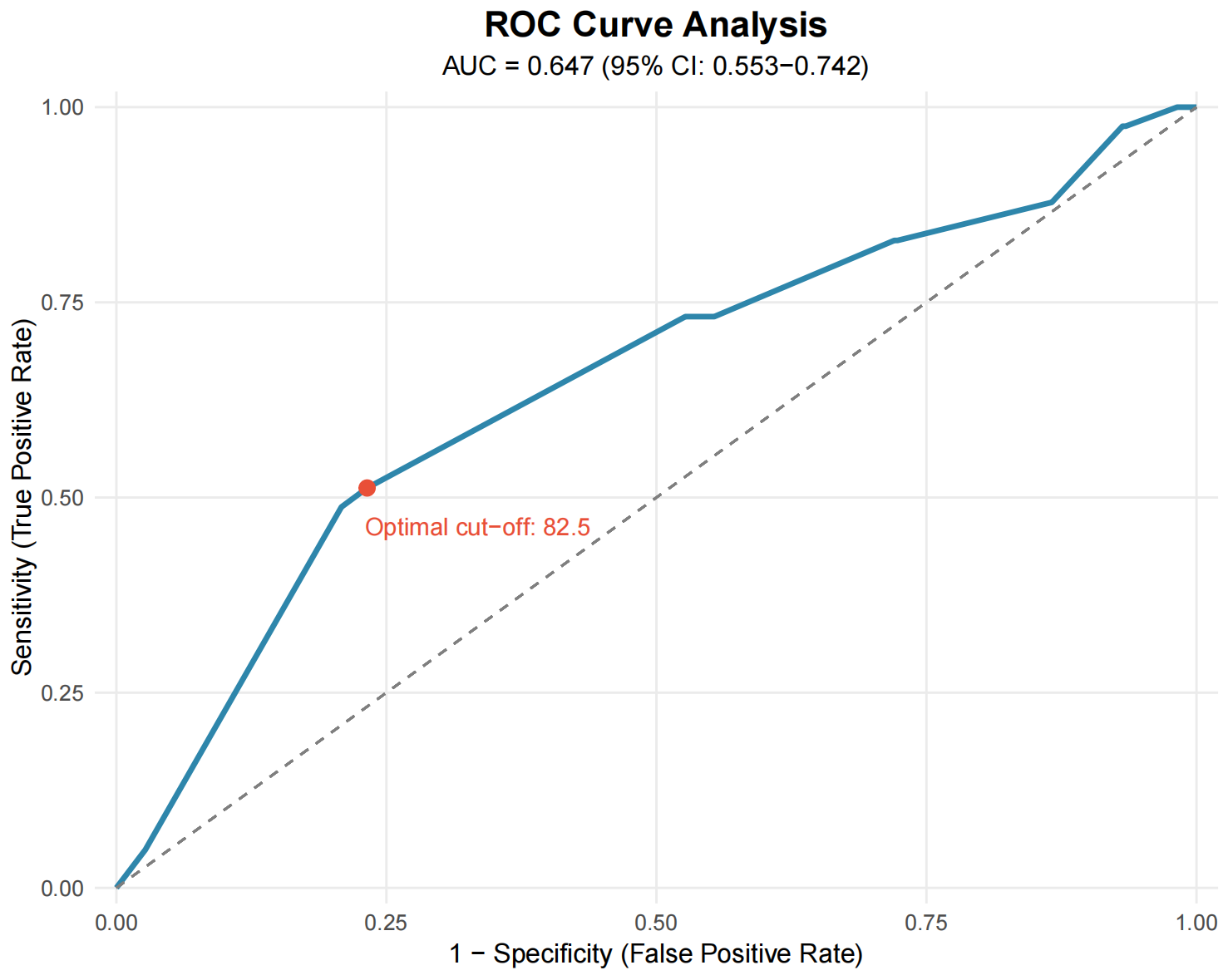

Supplement: S1 Fig — (TIF) [file pone.0350024.s001.tif]

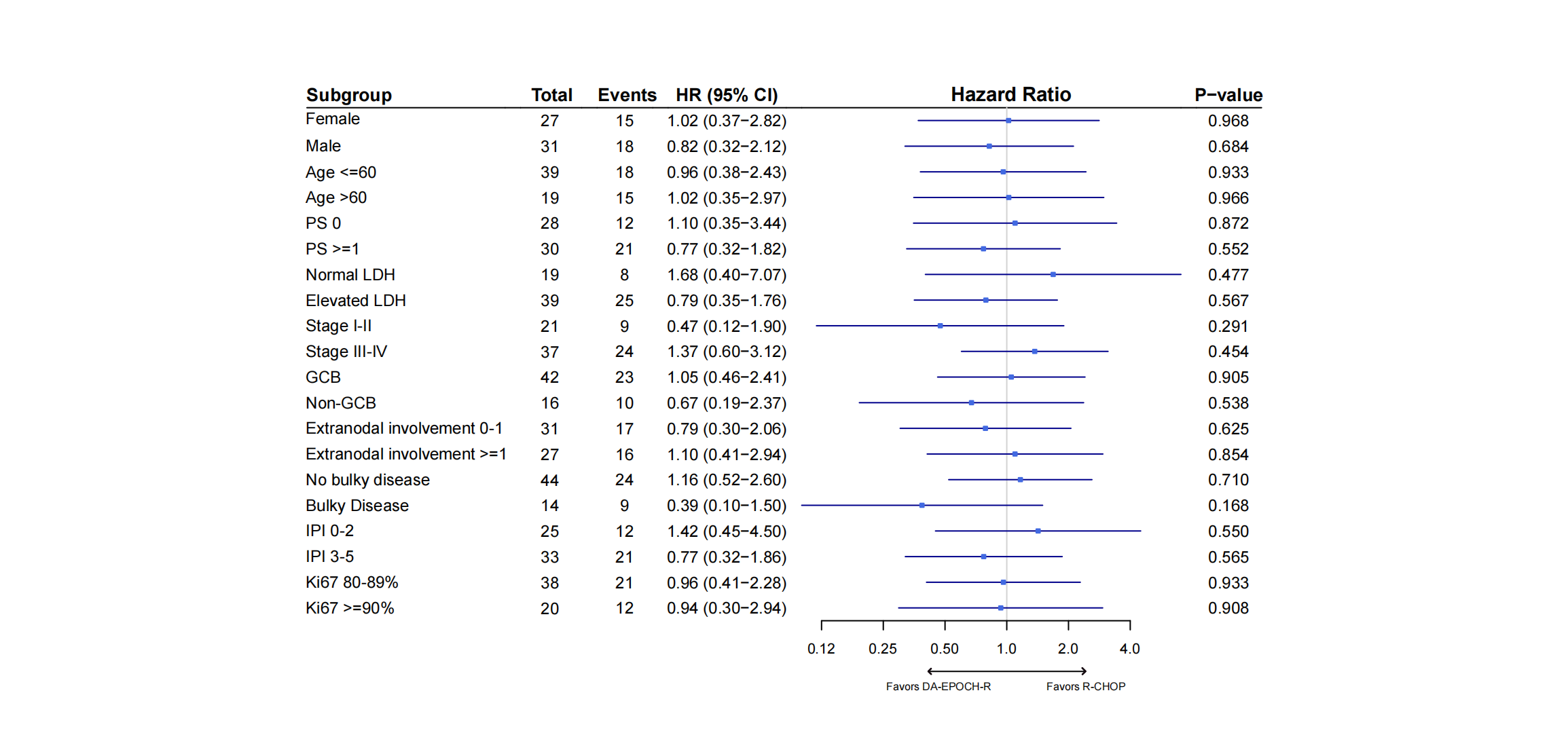

Supplement: S2 Fig — (TIF) [file pone.0350024.s002.tif]
