# Supplementary material for: Comparison of dose-adjusted EPOCH-R and R-CHOP in diffuse large B-cell lymphoma with high Ki67 expression: Results from a prospective observational study
Source: PLoS One. 2026 May 22;21(5):e0350024. doi: 10.1371/journal.pone.0350024 (PMC13196921; doi:10.1371/journal.pone.0350024)
Supplement: S1 Table — (DOCX) [file pone.0350024.s003.docx]

**S1 Table. Baseline characteristics of the retrospective cohort**

|  | number |
| --- | --- |
| Gender, n (%) |  |
| Male | 192 (51.1) |
| Female | 184 (48.9) |
| Age, years, median (range) | 56 (19-89) |
| GCB, n (%) | 166 (44.1) |
| Extranodal involvement > 1, n (%) | 197 (52.4) |
| Stage, n (%) |  |
| I-II | 243 (64.6) |
| III-IV | 133 (35.4) |
| ECOG PS, n (%) |  |
| 0 | 181 (48.1) |
| ≥1 | 195 (51.9) |
| Bulky disease, n (%) | 55 (14.6) |
| LDH > normal range, n (%) | 127 (33.8) |
| IPI≥3, n (%) | 62 (16.5) |
| Radiotherapy, n (%) | 115 (30.6) |

GCB: germinal center B-cell-like; ECOG PS: Eastern Cooperative Oncology Group Performance Status; LDH: lactate dehydrogenase; IPI: international prognostic index;
